# Radar-Based Assessment of Sit-to-Stand Transitions as Digital Biomarkers of Pain and Physical Decline

**DOI:** 10.3390/s26092769

**Published:** 2026-04-29

**Authors:** Mehri Ziaee Bideskan, Nima Karbaschi, Hajar Abedi, Zahra Abbasi

**Affiliations:** 1 Department of Electrical and Software Engineering, Schulich School of Engineering, University of Calgary, Calgary, AB T2N 1N4, Canada; mehri.ziaeebideskan@ucalgary.ca (M.Z.B.); nima.karbaschi@ucalgary.ca (N.K.); 2 School of Electrical Engineering and Computer Science, The University of Queensland, Brisbane, QLD 4072, Australia

**Keywords:** frequency-modulated continuous-wave (FMCW) radar, functional mobility assessment, home-based monitoring, non-contact motion sensing, Sit-to-Stand analysis

## Abstract

Sit-to-stand (STS) transitions are clinically informative indicators of functional independence and are sensitive to compensatory strategies associated with physical decline and pain. This study presents a non-contact, non-visual framework for quantitative STS assessment using a 60 GHz frequency-modulated continuous-wave (FMCW) radar in a residential setting. We developed a signal-processing pipeline that converts intermediate-frequency radar data into range–time intensity (RTI) maps, tracks dominant torso motion, and extracts temporal, kinematic, and spectral features. Experiments were conducted across two sensing orientations (subject-facing and side-facing), five mounting heights (45–153 cm), and three execution speeds, with approximately 30 repeated cycles per condition. For normal non-compensated STS transitions, radar-derived metrics reflected expected biomechanical scaling: mean full-cycle duration decreased from 23.90 s (slow) to 13.95 s (medium) and 7.98 s (fast), while peak ascent velocity increased from 0.311 m/s to 0.358 m/s and dominant cadence increased from 0.0416 Hz to 0.125 Hz. Simulated abnormal transitions produced distinct and quantifiable deviations. Preparatory rocking introduced an additional oscillatory phase (mean rocking duration 2.36 s), prolonging the standing transition to 4.80 s and altering trajectory regularity. Across configurations, subject-facing mid-torso mounting provided the most continuous and separable STS signatures, whereas side-facing placement and extreme heights reduced effective radial motion or introduced clutter artifacts. These findings establish practical deployment guidelines and demonstrate that radar-derived STS metrics can serve as candidate digital biomarkers for unobtrusive, privacy-preserving detection of mobility decline, compensatory pain behaviors, and functional impairment in real-world home environments.

## 1. Introduction

Mobility and functional independence are fundamental determinants of health, autonomy, and overall quality of life in aging populations [1,2]. Declines in motor performance are among the earliest and most sensitive indicators of frailty, fall risk, and cognitive deterioration, contributing directly to the loss of independence and increased rates of institutionalization [1,3]. In neurodegenerative disorders such as Alzheimer’s disease (AD), impaired mobility not only reflects disease progression but also exacerbates secondary complications, including physical inactivity, chronic pain, and social withdrawal [4,5]. Pain is highly prevalent among individuals with AD but remains profoundly underrecognized due to impaired communication and cognitive decline [6,7,8,9]. Patients frequently express distress through nonverbal behaviors such as facial grimacing, vocalizations, or agitation, which are often misinterpreted as psychiatric symptoms, leading to inappropriate pharmacologic treatment and prolonged, unrelieved pain [7]. This underrecognition creates a self-perpetuating cycle in which untreated pain accelerates inactivity, frailty, and behavioral disturbances, while progressive AD pathology further disrupts motor control and proprioception, contributing to immobility and secondary pain [10,11]. Emerging evidence suggests that effective pain detection and management may mitigate neuropsychiatric symptoms and slow cognitive decline, identifying pain as a potentially modifiable driver of disease progression. Movement-based functional tasks offer an objective window into both physical and neurocognitive health. Among these, the Sit-to-Stand (STS) maneuver is a fundamental component of activities of daily living and a well-established indicator of lower-limb strength, balance, and overall mobility. Beyond its apparent simplicity, STS represents a complex, multi-phase coordination task that requires precise integration of neuromuscular and cognitive control [12]. Successful execution depends on the ability to generate and scale momentum, maintain postural stability, and adapt balance dynamically during the transition from sitting to standing [13]. Consequently, STS performance is highly sensitive to both physical and cognitive impairments and has been recognized as an early predictor of frailty, fall risk, and functional decline in older adults [14]. In individuals with dementia, including AD, STS performance is affected by disturbances in motor planning, problem solving, and movement programming. Compared with cognitively healthy older adults, those with dementia adopt compensatory strategies to counteract impaired control such as greater reliance on armrests or backward repositioning of the feet to facilitate momentum generation. These measurable adaptations reflect underlying deficits in executive and motor function that disrupt the temporal organization and coordination of the task. Similarly, chronic pain alters STS biomechanics by inducing asymmetrical force generation and prolonged stabilization phases [15,16,17]. Together, these objective and quantifiable deviations in STS execution serve as digital indicators of cognitive degradation, pain severity, and overall physical decline.

Despite its diagnostic value, current STS assessment methods remain constrained by practical and methodological limitations. Traditional tools such as the PAINAD, Abbey Pain Scale, and Doloplus-2 quantify pain-related behaviors, but these assessments are episodic, observer-dependent, and often lack specificity [18,19]. Movement-based scales such as MOBID-2 improve pain localization by analyzing motor responses during guided tasks; however, they still rely on intermittent clinical observation and cannot capture continuous changes in functional performance. Standard STS-based clinical tests, including the Five-Times Sit-to-Stand [20] and 30-Second Chair Stand [21], similarly provide only aggregate outcomes (e.g., total duration or repetition count), offering little insight into compensatory strategies or sub-phase dynamics. Wearable sensors combined with machine learning (ML) have enabled more detailed quantification of movement kinematics and kinetics [22], yet their dependence on user compliance, correct placement, and frequent maintenance limits feasibility in frail or cognitively impaired populations. Vision-based approaches, including RGB and depth cameras, provide high-fidelity motion data but introduce major privacy concerns and are sensitive to lighting conditions and line-of-sight occlusion [23]. Collectively, these limitations highlight the need for a non-contact, privacy-preserving sensing modality capable of continuously and unobtrusively capturing fine-grained biomechanical information in real-world environments without requiring active user participation.

Millimeter-wave radar has emerged as a promising non-contact sensing modality for human motion analysis and healthcare monitoring, offering privacy-preserving operation and robustness to environmental variability such as lighting, clutter, and clothing [24,25,26]. By transmitting radio-frequency signals and analyzing their reflections, radar systems can capture range, velocity, and micro-motion information associated with human movement without recording identifiable visual data. This non-visual operation is particularly advantageous in privacy-sensitive environments such as bathrooms, bedrooms, and care facilities, where continuous camera-based monitoring is unacceptable. Recent advances in frequency-modulated continuous-wave (FMCW) and mmWave radar technology have enabled increasingly detailed characterization of functional activities. Abedi et al. [24] developed an AI-powered system for continuous in-home gait monitoring and activity recognition using FMCW radar integrated with cloud computing, demonstrating the feasibility of unobtrusive long-term monitoring in naturalistic environments. Building on this, the same group demonstrated non-contact multi-person gait monitoring in hallway environments [27] and continuous in-home gait analysis under naturalistic conditions [28], establishing radar as a viable modality for longitudinal mobility assessment. Fard et al. [29] demonstrated fall detection in ambient-assisted living environments using FMCW radar combined with deep learning, while Mishra and Pachori [30] applied higher-order synchrosqueezing transforms to FMCW radar signals for human activity recognition, and Gharamohammadi et al. [26] demonstrated radar integration into smart furniture for cardiac health monitoring, collectively illustrating the broad applicability of radar sensing across healthcare domains. Wang et al. [31] demonstrated that a 77 GHz FMCW radar can reliably extract six biomechanical gait parameters including step time, stride time, step length, stride length, torso velocity, and toe velocity, validated against a gold-standard motion capture system with average errors below 0.004 s in the time dimension and 0.002 m in the range dimension, further establishing the precision of contactless radar-based biomechanical parameter extraction in indoor environments.

Despite this progress, radar-based analysis of STS transitions specifically remains limited. Saho et al. [32] demonstrated that Doppler radar signatures of combined sit-to-stand and stand-to-sit movements can support accurate person identification, confirming that these transitions produce distinctive radar signatures. Notably, while radar signals can support movement-based person identification, this relies on kinematic signatures rather than visual appearance, preserving privacy in the visual sense while enabling functional monitoring. More recently, Hu et al. [33] conducted a comparative study of mmWave radar for STS analysis against Kinect and wearable sensors across 45 participants, using deep learning-based pose estimation and inverse kinematics to extract joint-level features. While their approach demonstrated excellent agreement for STS duration (Intraclass Correlation Coefficient, ICC = 0.96) and trunk peak velocity (ICC = 0.87) against reference sensors, it relies on complex skeleton reconstruction and a deep learning model trained on Kinect ground truth, limiting its applicability in privacy-sensitive or resource-constrained settings. In contrast, the present study adopts a signal-processing approach based directly on range–time intensity maps, requiring no visual ground truth, no skeleton model, and no deep learning infrastructure.

The present study investigates radar-based STS sensing from a systems-design and feasibility perspective, with three primary contributions. First, we propose a complete signal-processing pipeline that converts raw 60 GHz FMCW radar data into range–time intensity maps and extracts interpretable temporal, kinematic, and spectral features characterizing STS motion. Second, we provide design guidance for practical radar deployment by systematically evaluating the effects of sensing orientation and mounting height on signal clarity and feature extraction reliability, identifying subject-facing mid-torso placement as the most effective configuration. Third, we demonstrate that radar-derived features are sensitive to clinically relevant movement variations. For natural STS transitions, the extracted metrics exhibit systematic biomechanical scaling across execution speeds, with peak ascent velocity ranging from 0.311 m/s to 0.358 m/s and full-cycle duration from 23.90 s to 7.98 s. Simulated abnormal transitions produce distinct and quantifiable deviations, including preparatory rocking (mean rocking duration 2.36 s) and altered ascent dynamics associated with knee push-off. Experiments were conducted with a single healthy adult participant performing approximately 30 repeated STS cycles per condition, establishing proof-of-concept validation under controlled residential conditions. To assess generalizability, four additional healthy adults (height: 158–195 cm, weight: 45–75 kg) performed STS transitions at natural and slow execution speeds, with radar-extracted full-cycle durations confirming consistent speed discrimination across all participants. While population-level generalization requires future evaluation across diverse and clinically impaired cohorts, these findings demonstrate the feasibility of radar-derived STS metrics as candidate digital biomarkers for privacy-preserving, continuous mobility monitoring in real-world home environments.

## 2. Methodology

**Figure 1:**
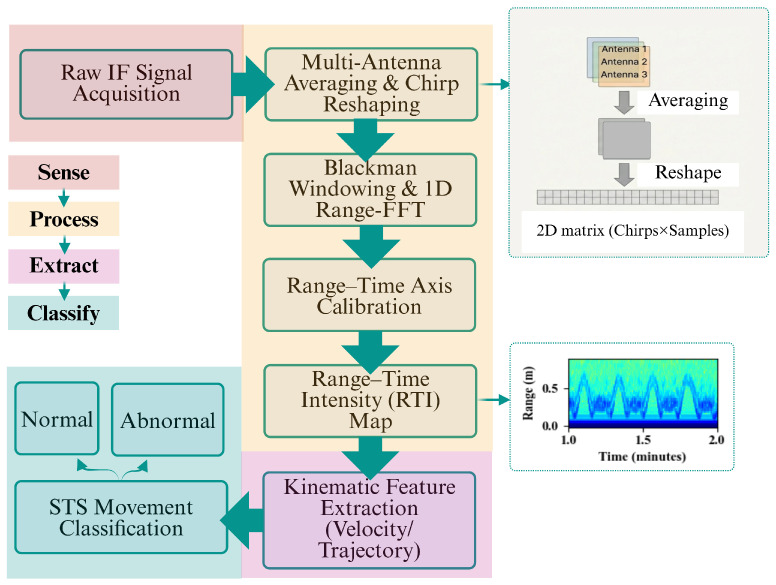
Overview of the radar-based STS sensing and classification pipeline.

This section outlines the radar signal processing framework and feature extraction strategy used to quantify STS motion. An overview of the proposed processing pipeline is illustrated in Figure 1.

### 2.1. Radar Signal Processing Pipeline

Raw intermediate-frequency (IF) radar data were acquired as a four-dimensional array x(n,c,m,k) indexed by frame *n*, receiver channel *c*, chirp *m*, and fast-time sample *k*. To improve the signal-to-noise ratio and mitigate channel-dependent variability, the received signals were averaged across the *C* receiver antennas as

(1) $$\bar{x}\left(n,m,k\right)=\frac{1}{C}\sum_{c=1}^{C}x\left(n,c,m,k\right).$$

The averaged data were reshaped into a two-dimensional matrix in which each row corresponds to a single chirp over time. Range information was then obtained by applying a one-dimensional fast Fourier transform (FFT) along the fast-time dimension. Prior to the FFT, a Blackman window w(k) was applied to suppress spectral leakage and reduce sidelobes:

(2) $$X\left(n,m,r\right)=\sum_{k=0}^{N-1}\bar{x}\left(n,m,k\right)\;w\left(k\right)\;e^{-j2\pi rk/N}.$$

where *N* denotes the number of fast-time samples per chirp and *k* is the corresponding sample index. In the implementation, the chirp dimension is neither averaged nor discarded. Each chirp within each frame is treated as an independent slow-time snapshot. The three-dimensional array $\bar{x}\left(n,m,k\right)$ (after antenna averaging) was reshaped by unrolling the frame index *n* and chirp index *m* into a single linear slow-time index:

(3) t=n·M+m,

where M=64 is the number of chirps per frame. The corresponding physical time is:

(4) $$t_{\mathrm{phys}}=\frac{t}{M}\cdot T_{\mathrm{frame}},$$

where $T_{\mathrm{frame}}=77.27$ ms is the frame repetition time. This yields a uniform slow-time axis with an effective temporal resolution equal to the chirp repetition time $T_{\mathrm{chirp}}=T_{\mathrm{frame}}/M\approx 1.207$ ms per snapshot. The Blackman-windowed range FFT was then applied along the fast-time dimension of this reshaped matrix, and only the one-sided positive frequency spectrum was retained. The magnitude-squared FFT output was converted to logarithmic scale to form the range–time intensity (RTI) map:

(5) $$\mathrm{RTI}\left(t,r\right)=10\log_{10}\left({\left|X\left(t,r\right)\right|}^{2}\right),$$

where *t* denotes the slow-time index obtained from consecutive chirps and frames. The physical range axis was derived from the beat-frequency bins using the FMCW chirp slope *S*, computed from the effective bandwidth *B* and analog-to-digital converter (ADC) sampling period $T_{\mathrm{ADC}}$. The corresponding range is given by

(6) $$R=\frac{c\;f_{b}}{2S},\;S=\frac{B}{T_{\mathrm{ADC}}},$$

where *c* is the speed of light and $f_{b}$ is the beat frequency. The time axis was constructed by accounting for both the chirp repetition interval and frame delays, ensuring an accurately scaled temporal representation. This processing framework yields RTI maps that represent the dominant radial displacement of the subject’s torso over time, enabling subsequent extraction of temporal, kinematic, and spectral features associated with STS transitions.

### 2.2. STS Feature Extraction

The proposed feature extraction framework comprises four categories as summarized in Table 1: temporal, kinematic, spectral, and behavioral. Temporal features (Equations (7)–(9)) characterize the global timing structure of the STS task and are adapted from established clinical STS assessment metrics. Kinematic features (Equations (10)–(17)) quantify the radial displacement and velocity of the torso trajectory and are derived from the radar range signal using standard differentiation. Spectral features (Equations (18)–(20)) characterize the rhythmic structure and cadence of repeated STS cycles and represent a radar-specific contribution, exploiting the frequency content of the range trajectory in a manner not previously applied to STS assessment. Behavioral features (Equations (21)–(26)) quantify temporal sub-phases of abnormal transitions including preparatory rocking, as described in Section 4.2.4.

#### 2.2.1. Temporal Feature Extraction

STS motion was quantified using a set of features extracted from the RTI maps. Temporal characteristics of the STS motion were extracted from the smoothed range trajectory derived from the RTI maps. The dominant torso trajectory was first identified by tracking the maximum-power range bin at each time instant, yielding a one-dimensional range profile that represents the subject’s radial displacement over time. To ensure a continuous and physically plausible trajectory, range-bin migration artifacts were suppressed by constraining sudden inter-sample range variations exceeding a predefined threshold to the previously validated torso position. The resulting 1D range trajectory was subsequently smoothed using a Savitzky–Golay filter with window length W=1001 samples and polynomial order 3.

Seated and standing positions were detected using dual empirical range thresholds, denoted by $\theta_{\mathrm{sit}}$ and $\theta_{\mathrm{stand}}$, corresponding to the maximum-range (seated) and minimum-range (standing) torso locations, respectively. Rather than using the argmax of the seated plateau as a landmark, which introduces cycle-to-cycle jitter since the peak can land anywhere within the flat plateau region, two deterministic edge points are defined for each seated event: the left edge $t_{\mathrm{le},i}$, corresponding to the first sample where the smoothed trajectory exceeds $\theta_{\mathrm{sit}}$, marking the moment the subject fully settles into the seat; and the right edge $t_{\mathrm{re},i}$, corresponding to the last sample where the smoothed trajectory exceeds $\theta_{\mathrm{sit}}$, marking the moment the subject begins to rise. The standing valley $t_{v,i}$ is defined as the time instant of minimum range in the smoothed trajectory below $\theta_{\mathrm{stand}}$ between two consecutive seated plateaus. A full cycle was defined from one seated left edge to the subsequent seated left edge.

Based on these detected events, three temporal parameters were defined for each cycle. The full cycle duration was computed as

(7) $$T_{\mathrm{cycle}}\left(i\right)=t_{\mathrm{le},i+1}-t_{\mathrm{le},i},$$

representing the total time between two consecutive seated left-edge landmarks, corresponding to the moment the subject fully settles into the seat at the start of each cycle.

The ascent duration, corresponding to the sit-to-stand transition, was defined as

(8) $$T_{\mathrm{ascent}}\left(i\right)=t_{\mathrm{le},i}-t_{v,i},$$

representing the time interval from the standing valley $t_{v,i}$ to the seated left-edge landmark $t_{\mathrm{le},i}$, capturing the active rising phase of the STS transition.

The descent duration, corresponding to the stand-to-sit transition, was defined as

(9) $$T_{\mathrm{descent}}\left(i\right)=t_{v,i}-t_{\mathrm{re},i},$$

representing the time interval from the seated right-edge landmark $t_{\mathrm{re},i}$, marking the moment the subject begins to rise, to the subsequent standing valley $t_{v,i}$, capturing the active lowering phase of the STS transition. These temporal parameters characterize both the global cadence of the STS task and the relative timing of its constituent phases.

#### 2.2.2. Kinematic Feature Extraction

Radial kinematic features were subsequently derived from the smoothed range trajectory. The instantaneous radial velocity was computed as the first temporal derivative of the reconstructed range signal

(10) $$v\left(t\right)=\frac{dR(t)}{dt},$$

where R(t) denotes the smoothed dominant torso range trajectory.

For each STS cycle, velocity and displacement features were computed separately for the ascent and descent phases defined by the detected standing and seated events. The displacement during the ascent phase was computed as

(11) $$D_{\mathrm{ascent}}\left(i\right)=R\left(t_{\mathrm{le},i}\right)-R\left(t_{v,i}\right),$$

representing the radial displacement from the standing valley $t_{v,i}$ to the seated left-edge landmark $t_{\mathrm{le},i}$, corresponding to the torso displacement during the rising phase of the STS transition. Similarly, the displacement during the descent phase was computed as

(12) $$D_{\mathrm{descent}}\left(i\right)=R\left(t_{v,i}\right)-R\left(t_{\mathrm{re},i}\right),$$

representing the radial displacement from the seated right-edge landmark $t_{\mathrm{re},i}$ to the subsequent standing valley $t_{v,i}$, corresponding to the torso displacement during the lowering phase of the STS transition. Average and peak velocities were then extracted for each phase. The mean ascent velocity was defined as

(13) $${\bar{v}}_{\mathrm{ascent}}\left(i\right)=\frac{D_{\mathrm{ascent}}\left(i\right)}{T_{\mathrm{ascent}}\left(i\right)},$$

and the mean descent velocity was defined as

(14) $${\bar{v}}_{\mathrm{descent}}\left(i\right)=\frac{D_{\mathrm{descent}}\left(i\right)}{T_{\mathrm{descent}}\left(i\right)}.$$

The peak ascent and descent velocities were computed as

(15) $$v_{\mathrm{ascent}}^{\mathrm{peak}}\left(i\right)=\max_{t\in [t_{v,i},\;t_{\mathrm{le},i}]}\dot{R}\left(t\right),$$

(16) $$v_{\mathrm{descent}}^{\mathrm{peak}}\left(i\right)=\min_{t\in [t_{\mathrm{re},i},\;t_{v,i}]}\dot{R}\left(t\right),$$

corresponding to the maximum positive velocity during ascent and the maximum negative velocity during descent, respectively. To characterize motion asymmetry between the rising and lowering phases, a velocity ratio feature was additionally defined as

(17) $$\rho_{v}=\left|\frac{\bar{v}\mathrm{ascent}}{\bar{v}\mathrm{descent}}\right|.$$

#### 2.2.3. Spectral Feature Extraction

To further characterize the rhythmic structure of the STS motion, spectral features were extracted from the dominant torso range trajectory to characterize the rhythmic structure of the STS motion. The smoothed range signal R(t) was transformed into the frequency domain using an FFT,

(18) X(f)=F{R(t)}.

The dominant STS cycle frequency was defined as the frequency corresponding to the maximum spectral magnitude

(19) $$f_{\mathrm{peak}}=\arg\max_{f}\left|X\left(f\right)\right|,$$

which represents the fundamental repetition rate of the STS motion. The corresponding cycle period was obtained as $T_{\mathrm{cycle}}=1/f_{\mathrm{peak}}$.

To quantify the spectral concentration of the movement, a bandwidth measure was additionally computed at the −3 dB level relative to the dominant spectral peak. The effective bandwidth was defined as

(20) $$BW_{3\;\mathrm{dB}}=f_{r}-f_{l},\;\mathrm{such}\;\mathrm{that}\;|X\left(f_{l,r}\right)|=\frac{1}{\sqrt{2}}\left|X\left(f_{\mathrm{peak}}\right)\right|,$$

where $f_{l}$ and $f_{r}$ denote the lower and upper frequency bounds at which the spectral magnitude drops to $1/\sqrt{2}$ of the peak value.

Together, $f_{\mathrm{peak}}$ and $BW_{3\;\mathrm{dB}}$ provide compact spectral descriptors of STS cadence and movement regularity, with higher peak frequencies corresponding to faster execution speeds and narrower bandwidths indicating more periodic and stable motion patterns.

#### 2.2.4. Abnormal STS Transition Modeling

In addition to natural STS motions, a set of simulated abnormal transitions was recorded to capture compensatory behaviors commonly observed in individuals with mobility impairments. These abnormal patterns were intentionally performed under controlled conditions and included: (i) pushing off the knees during the sit-to-stand phase, (ii) preparatory rocking prior to standing, and (iii) motion speed variability across consecutive repetitions.

Among the simulated abnormal conditions, preparatory rocking prior to standing required additional temporal characterization. The same event detection strategy described for natural STS transitions was applied, where global maxima $t_{p,i}$ correspond to seated positions and global minima $t_{v,i}$ correspond to fully standing positions.

The full cycle duration for rocking trials was defined consistently with the normal case as the time between two consecutive fully standing positions

(21) $$T_{\mathrm{cycle}}^{\mathrm{rock}}\left(i\right)=t_{v,i+1}-t_{v,i}.$$

Within each cycle, multiple seated peaks were observed between two consecutive standing valleys. Let $t_{p,i}^{\left(1\right)}$ denote the first seated peak after $t_{v,i}$ and $t_{p,i}^{\left(k\right)}$ denote the final seated peak preceding $t_{v,i+1}$.

The descent duration was defined as the time from the fully standing position to the first seated peak within the same cycle

(22) $$T_{\mathrm{descent}}^{\mathrm{rock}}\left(i\right)=t_{p,i}^{\left(1\right)}-t_{v,i}.$$

The rocking duration, representing the preparatory forward–backward oscillations, was defined as the time interval between the first and final seated peaks within the rocking window

(23) $$T_{\mathrm{rock}}\left(i\right)=t_{p,i}^{\left(k\right)}-t_{p,i}^{\left(1\right)}.$$

The final ascent duration was defined as the time from the last seated peak to the subsequent fully standing position

(24) $$T_{\mathrm{ascent}}^{\mathrm{rock}}\left(i\right)=t_{v,i+1}-t_{p,i}^{\left(k\right)}.$$

For completeness, a standing transition duration was also computed as

(25) $$T_{\mathrm{stand}}\left(i\right)=t_{v,i+1}-t_{p,i}^{\left(1\right)},$$

which, by definition, satisfies

(26) $$T_{\mathrm{stand}}\left(i\right)=T_{\mathrm{rock}}\left(i\right)+T_{\mathrm{ascent}}^{\mathrm{rock}}\left(i\right).$$

These additional temporal subdivisions allow explicit quantification of preparatory oscillations and differentiate rocking behavior from natural STS transitions while maintaining consistency with the previously defined event framework.

## 3. Experimental Setup

Experiments were conducted using a healthy adult participant (height: 161 cm) performing repeated STS motions in a residential indoor environment. The study design focused on feasibility and system characterization rather than population-level inference. All measurements were performed without wearable sensors or physical markers to emulate zero-effort, real-world monitoring conditions.

A 60-GHz FMCW radar sensor (BGT60TR13C, Infineon Technologies AG, Neubiberg, Germany) was used to acquire radar data. The sensor was configured with one transmit and three receive antennas. Radar data acquisition was performed using the Infineon Radar SDK version 3.6.4 (Infineon Technologies AG, Neubiberg, Germany), and subsequent signal processing and feature extraction were implemented in Python version 3.11. Detailed radar configuration parameters are summarized in Table 2. The radar sensor operates at a transmit power well below the regulatory limits established by FCC and ETSI for unlicensed millimeter-wave devices [34], and emits non-ionizing radiation at a specific absorption rate negligible at the operating distances used in this study [35], confirming the safety of the system for close-proximity human monitoring applications.

**Figure 2:**
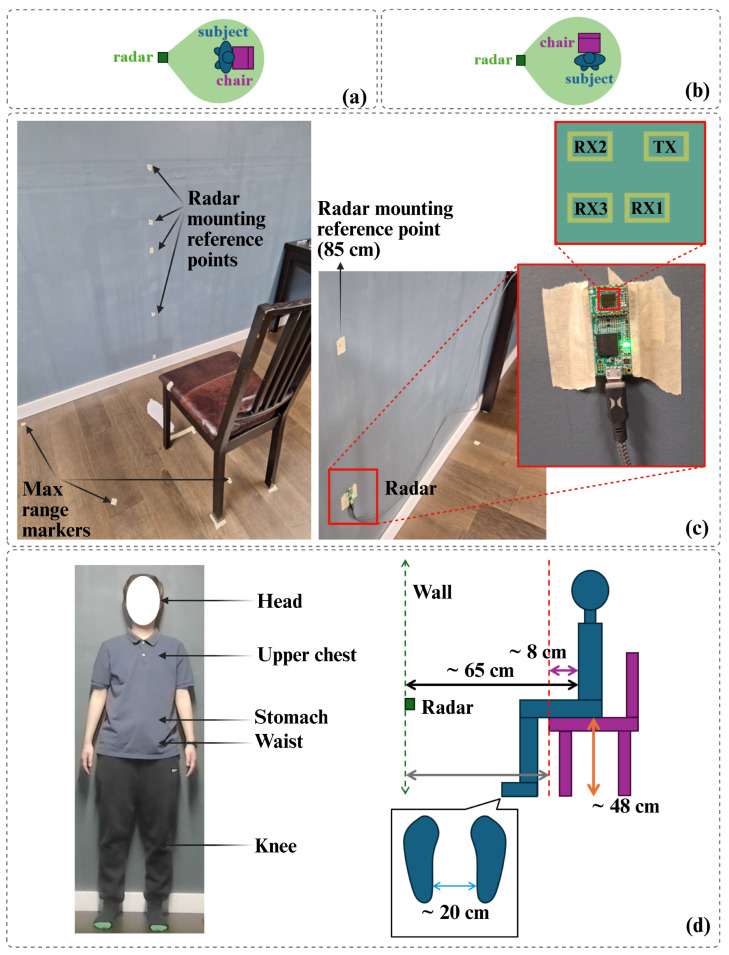
Experimental setup and measurement geometry. (**a**) Subject-facing radar (SFR) configuration and (**b**) side-facing radar (SSFR) configuration. (**c**) Measurement environment showing radar mounting reference points and spatial alignment markers for consistent positioning. (**d**) Anatomical reference heights used for radar placement (knee, waist, stomach, upper chest, and head). (**left**) and schematic of the subject–chair–radar geometry with key distances and dimensions (**right**).

The radar was evaluated under two placement geometries: a subject-facing configuration (SFR), positioned directly in front of the participant, and a side-facing configuration (SSFR), positioned laterally. For each geometry, the radar was mounted at five anatomically relevant heights—knee (45 cm), waist (85 cm), stomach (100 cm), upper chest (125 cm), and head (153 cm)—selected to reflect realistic integration into everyday furniture and living environments. The relative positions of the radar, chair, and subject were kept constant across all trials. A detailed illustration of the experimental environment, including radar placement geometries, anatomical mounting heights, and spatial alignment between the radar, chair, and subject, is shown in Figure 2.

The participant performed approximately 30 repeated STS cycles at three controlled movement speeds: slow, medium, and fast. These speeds corresponded to full STS cycle durations of approximately 24 s, 14 s, and 8 s, respectively, and were selected based on fall-risk–related thresholds reported in prior clinical literature. Each repetition consisted of a complete STS cycle, including quiet sitting, trunk flexion, lift-off, ascent, and postural stabilization. In addition to natural STS motions, abnormal transitions were deliberately performed to emulate compensatory strategies and movement irregularities commonly observed in mobility-impaired populations, including reliance on upper-limb assistance, preparatory rocking, and increased cycle-to-cycle variability.

## 4. Results and Discussion

This section presents and interprets the experimental findings obtained from radar-based monitoring of STS transitions under diverse sensing configurations and dynamic movement conditions. The results are organized to evaluate: (i) the influence of radar orientation and mounting height on the quality and morphology of range–time signatures; (ii) the variability of extracted temporal, kinematic, and spectral features across different movement speeds and transition types; and (iii) the ability of these features to distinguish between normal and abnormal STS executions. Collectively, these analyses elucidate the sensing geometry and signal characteristics that maximize the reliability of radar-derived STS metrics for functional mobility assessment.

### 4.1. Effect of Radar Orientation and Height on STS Signatures

**Figure 3:**
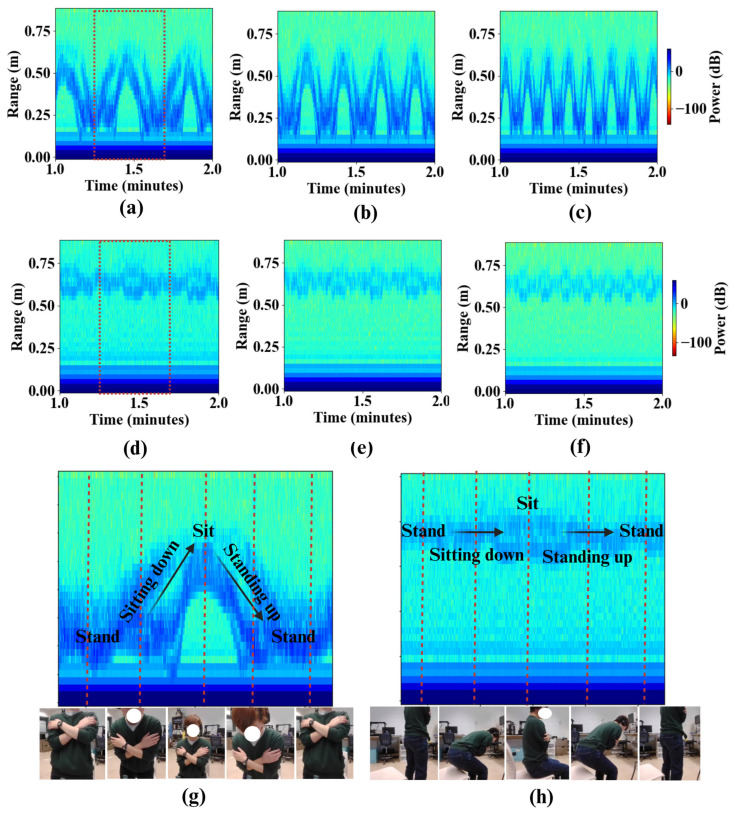
RTI maps of STS motion recorded with the radar mounted at waist height (85 cm). (**a**) SFR configuration at slow speed. (**b**) SFR configuration at medium speed. (**c**) SFR configuration at fast speed. (**d**) SSFR configuration at slow speed. (**e**) SSFR configuration at medium speed. (**f**) SSFR configuration at fast speed. (**g**) Zoomed representative STS cycle in the SFR configuration with annotated motion phases. (**h**) Zoomed representative STS cycle in the SSFR configuration with annotated motion phases.

Figure 3 compares the RTI maps obtained using the SFR and SSFR configurations during repeated STS motions performed at slow, medium, and fast speeds. In the SFR configuration (top row), each STS cycle produces a distinct V-shaped trajectory, corresponding to the subject’s downward motion during sitting and upward motion during standing. These trajectories exhibit strong range modulation and clear temporal periodicity across all tested speeds, indicating high sensitivity to vertical trunk displacement.

In contrast, the SSFR configuration (middle row) yields substantially weaker range variation and reduced trajectory contrast. Although periodic motion remains observable, the characteristic V-shaped pattern becomes less pronounced and appears compressed along the range axis. This difference arises from the change in viewing geometry: in the SFR case, the radar line-of-sight aligns with the dominant anterior–posterior displacement of the torso during STS, whereas in the SSFR case the motion occurs largely orthogonal to the radar beam, resulting in a reduced radial displacement component.

The annotated examples in the bottom row further illustrate the segmentation of individual STS phases—standing, sitting down, sitting, and standing up—in both configurations. While phase transitions remain detectable in SSFR, the SFR configuration provides more clearly separable phase boundaries and stronger signal modulation. These results demonstrate that radar orientation has a substantial impact on the morphology and clarity of STS signatures, with subject-facing placement providing more reliable and interpretable motion representations for subsequent feature extraction.

**Figure 4:**
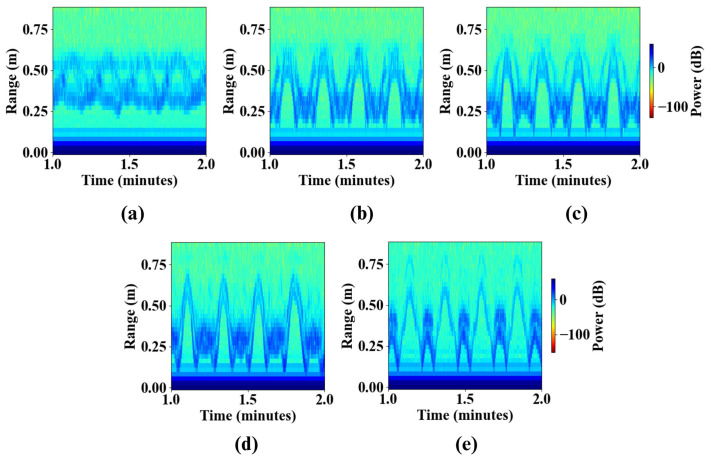
Effect of radar mounting height on the RTI signatures of STS motion in the SFR configuration. RTI maps are shown for five anatomically relevant heights ((**a**) knee, (**b**) waist, (**c**) stomach, (**d**) upper chest, and (**e**) head).

The influence of radar mounting height is primarily reflected in the clarity and stability of the extracted torso trajectory in the RTI maps. As shown in Figure 4, intermediate mounting height (waist) produce the most distinct and continuous V-shaped signatures, corresponding to the radial displacement of the torso during STS transitions. At this height, the radar beam is aligned with the largest reflective body surface, maximizing signal-to-noise ratio and yielding a well-defined trajectory suitable for temporal and kinematic feature extraction.

In contrast, lower mounting positions (e.g., knee height) result in weaker and noisier signatures due to increased sensitivity to leg motion and stronger ground-plane reflections, which appear as persistent high-intensity bands at short ranges. Higher mounting positions reduce floor clutter but introduce partial shadowing effects as the head and shoulders occlude the torso during the ascent phase, leading to intermittent signal attenuation near the peak of the motion.

These observations indicate that radar placement near the torso center of mass provides the most reliable representation of STS kinematics by balancing signal strength, clutter suppression, and trajectory continuity.

### 4.2. Temporal, Kinematic, and Spectral Feature Analysis

#### 4.2.1. Temporal Features

**Figure 5:**
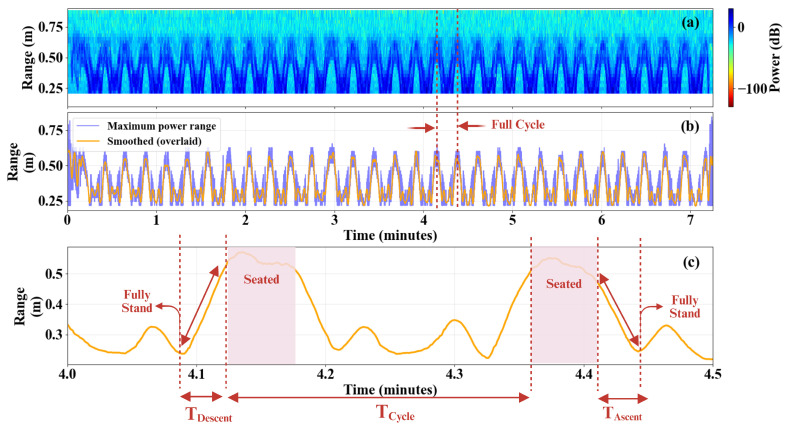
Temporal feature extraction from waist-mounted radar measurements during 30 repeated medium-speed STS transitions. (**a**) Stationary-filtered RTI map showing consecutive STS transitions. (**b**) One-dimensional maximum-power range profile (blue) and the Savitzky–Golay smoothed trajectory (orange), with a representative full cycle indicated. (**c**) Magnified segment of the smoothed trajectory illustrating the definition of temporal parameters: ascent duration ($T_{\mathrm{ascent}}$), descent duration ($T_{\mathrm{descent}}$), and full cycle duration ($T_{\mathrm{cycle}}$).

Temporal feature extraction from waist-mounted radar measurements during repeated STS trials is illustrated in Figure 5. Figure 5a shows the stationary-filtered RTI map for 30 consecutive medium-speed STS transitions. The corresponding one-dimensional maximum-power range profile and the reconstructed smoothed trajectory are shown in Figure 5b. A magnified segment of the smoothed trajectory is presented in Figure 5c to visualize the temporal markers defined in Section 3.

These results correspond to waist-mounted radar measurements acquired at medium execution speed. The same processing and feature extraction procedure was applied to slow, medium, and fast STS trials. For each speed condition, the mean and standard deviation of the temporal parameters $T_{\mathrm{cycle}}$, $T_{\mathrm{ascent}}$, and $T_{\mathrm{descent}}$ were computed across 30 repeated cycles and are summarized in Table 3.

The extracted temporal features exhibit a clear and systematic dependence on execution speed. As shown in Table 3, the mean full-cycle duration $T_{\mathrm{cycle}}$ decreases monotonically from 23.90 s in the slow condition to 13.95 s at medium speed and 7.98 s at fast speed. These values are consistent with the experimentally prescribed STS cycle durations of approximately 12 s, 7 s, and 4 s per transition phase, corresponding to full-cycle periods of roughly 24 s, 14 s, and 8 s, respectively. This agreement confirms that the proposed radar-based method accurately captures the global timing structure of the STS motion.

Similarly, both ascent and descent durations decrease proportionally with increasing speed, indicating that the extracted features preserve the relative temporal scaling of the underlying biomechanical phases. The low standard deviations observed across the 30 repetitions reflect the combined consistency of the participant’s movement execution and the stability of the radar-based feature extraction pipeline. While these two sources of variability cannot be separated without a concurrent ground-truth reference, the agreement between the extracted mean cycle durations and the intended execution speeds ($T_{\mathrm{cycle}}\approx 24$ s, 14 s, and 8 s for slow, medium, and fast conditions respectively) supports the capacity of the proposed pipeline to track clinically relevant STS timing metrics across a range of movement speeds. Collectively, these results validate the use of radar-derived temporal parameters as reliable surrogates for clinically relevant STS timing metrics.

**Figure 6:**
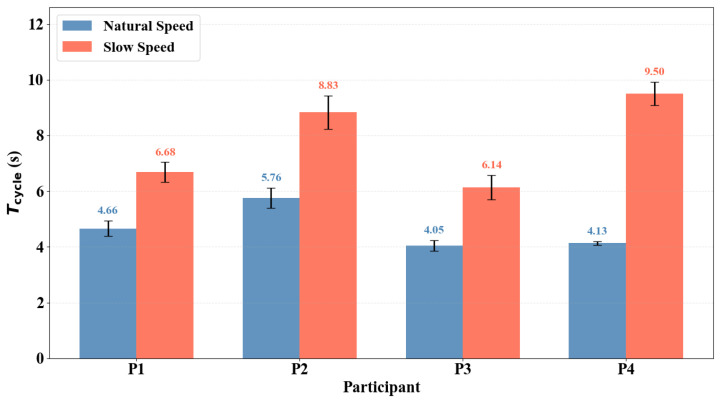
Radar-extracted mean full-cycle duration $T_{\mathrm{cycle}}$ for four participants at natural and slow execution speeds. Error bars represent one standard deviation across cycles.

To assess the generalizability of the proposed pipeline across participants, four additional healthy adults were recruited (two females, two males; height: 158–195 cm, weight: 45–75 kg). Each participant performed 15 repeated STS cycles at natural and slow execution speeds using the same waist-height SFR radar configuration as the original experiment. Figure 6 presents the radar-extracted mean full-cycle duration $T_{\mathrm{cycle}}$ for each participant at both speeds. The slow condition consistently produced larger $T_{\mathrm{cycle}}$ values than the natural condition for all four participants, with natural speed ranging from 4.05±0.19 s to 5.76±0.36 s and slow speed ranging from 6.14±0.44 s to 9.50±0.42 s across participants. This monotonic relationship was preserved regardless of participant height, weight, or movement style, confirming that the proposed radar-based pipeline generalizes across individuals with different body characteristics and movement patterns.

#### 4.2.2. Kinematic Features

**Figure 7:**
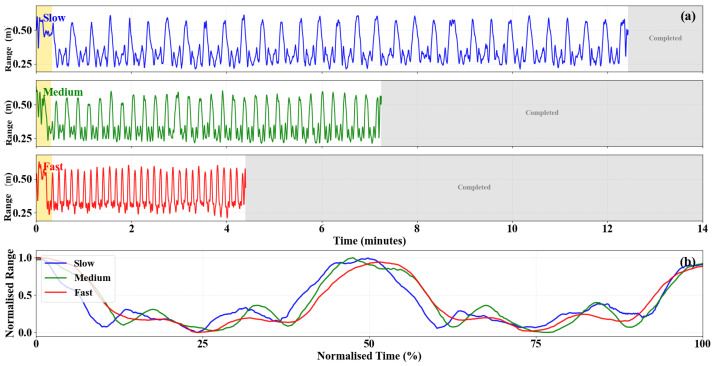
Kinematic trajectories of torso motion during repeated STS transitions at different execution speeds. (**a**) Savitzky–Golay smoothed range profiles extracted from RTI maps for slow (blue), medium (green), and fast (red) trials, each displayed on a shared time axis. The yellow shaded region indicates the preparation period prior to the first STS cycle. The gray shaded region indicates the period after completion of all 30 repetitions. (**b**) Two representative consecutive cycles for each speed condition, normalized to percentage of total duration and range, confirming that the fundamental trajectory shape is preserved across execution speeds and across consecutive repetitions.

Kinematic characteristics of the STS motion were quantified from the smoothed range trajectory by computing displacement- and velocity-based features for the ascent and descent phases. Figure 7 illustrates representative torso trajectories at slow, medium, and fast execution speeds, highlighting systematic changes in movement dynamics as speed increases.

Figure 7a shows the full recording duration for each speed condition, demonstrating that the 30 repeated STS cycles occupy progressively shorter total durations as execution speed increases, from approximately 12 min for the slow condition to approximately 7 min for the medium and 4.5 min for the fast condition. Figure 7b shows two representative consecutive cycles for each speed condition, normalized to percentage of total duration. The three curves follow a closely similar trajectory shape across both cycles, confirming that the fundamental biomechanical pattern of STS motion is preserved across execution speeds and across consecutive repetitions.

Table 4 summarizes the extracted kinematic features. The displacement during ascent ($D_{\mathrm{ascent}}$) and descent ($D_{\mathrm{descent}}$) remained approximately constant across execution speeds, with magnitudes close to 0.27–0.33 m. This consistency reflects the fixed geometric distance between seated and standing torso positions, indicating that variations in execution speed primarily affect movement dynamics rather than spatial excursion.

In contrast, clear speed-dependent trends were observed in the velocity features. The average ascent velocity ($V_{\mathrm{ascent},\mathrm{avg}}$) increased from 0.129±0.028 m/s in the slow condition to 0.212±0.072 m/s in the fast condition. A similar pattern was observed for the average descent velocity ($V_{\mathrm{descent},\mathrm{avg}}$), whose magnitude increased from 0.094±0.029 m/s to 0.237±0.083 m/s. Peak velocities exhibited the same monotonic trend, with $V_{\mathrm{ascent},\mathrm{peak}}$ rising from 0.311±0.070 m/s (slow) to 0.358±0.030 m/s (fast), and $V_{\mathrm{descent},\mathrm{peak}}$ increasing in magnitude from 0.305±0.098 m/s to 0.384±0.050 m/s.

The velocity ratio, defined as the magnitude ratio between average ascent and descent velocities, remained close to unity across all speeds (0.983–1.046), indicating a largely symmetric execution of the sit-to-stand and stand-to-sit phases in this healthy participant. This symmetry is also evident in the trajectories shown in Figure 7, where ascent and descent segments exhibit comparable slopes within each speed condition.

Overall, these results demonstrate that the proposed radar-based framework reliably captures both spatial and dynamic properties of STS motion. While displacement features remain stable across speeds, velocity-based features provide strong sensitivity to execution speed and therefore constitute informative kinematic biomarkers for assessing movement intensity and potential motor impairment.

It is noted that no concurrent reference modality was employed in this feasibility study. Indirect validation was performed through three complementary approaches: (i) agreement between radar-extracted cycle durations and experimentally prescribed movement timing (mean error below 0.8 s across all speed conditions); (ii) internal consistency of radar-derived torso displacement (0.27–0.33 m) and velocity ratio (0.983–1.046) across all speeds and 30 repeated cycles, confirming measurement stability and physical plausibility; and (iii) support from prior radar-based STS validation demonstrating excellent agreement between 60 GHz radar and reference sensors including Kinect and wearable inertial measurement units for STS duration (ICC =0.96) and trunk peak velocity (ICC =0.87) [31]. Cross-modal validation against inertial measurement units or optical motion capture is identified as a priority direction for future work.

#### 4.2.3. Spectral Features

The spectral characteristics of the STS motion were quantified using the dominant cycle frequency $f_{\mathrm{peak}}$ and the corresponding −3 dB bandwidth $BW_{3\;\mathrm{dB}}$, extracted from the frequency domain representation of the torso range trajectory.

As summarized in Table 5, the dominant frequency increased systematically with execution speed, rising from 0.0416 Hz for slow trials to 0.0712 Hz for medium trials and 0.1250 Hz for fast trials. This trend reflects the expected increase in movement cadence as participants performed STS transitions more rapidly.

In addition to the frequency shift, the spectral bandwidth also increased with speed, from 0.0027 Hz (slow) to 0.0076 Hz (fast). The broader bandwidth observed at higher speeds indicates greater variability and higher dynamic content in the movement pattern, consistent with reduced stability and increased neuromuscular demand during rapid transitions.

These spectral features provide a compact representation of STS rhythmicity and steadiness and complement the temporal and kinematic descriptors. In particular, $f_{\mathrm{peak}}$ characterizes the global cadence of the task, while $BW_{3\;\mathrm{dB}}$ reflects the regularity of repeated cycles, making them well suited for distinguishing between different execution speeds and potentially identifying abnormal or irregular movement strategies.

#### 4.2.4. Abnormal STS Transitions

**Figure 8:**
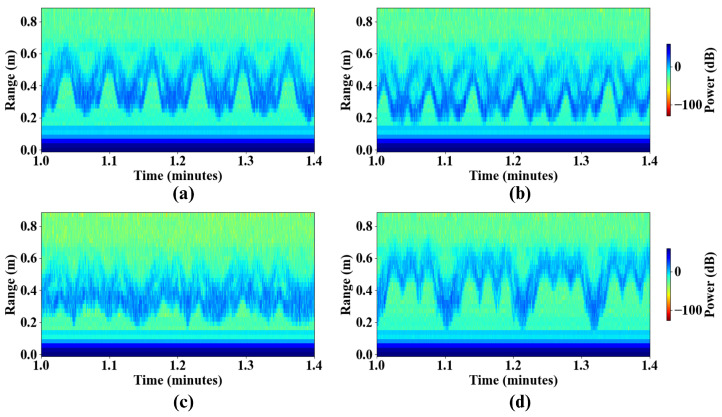
RTI maps of representative STS transitions under different movement conditions: (**a**) natural motion, (**b**) pushing-off-knees, (**c**) motion speed variability, and (**d**) preparatory rocking.

The RTI maps in Figure 8 illustrate representative STS transitions for normal and abnormal movement patterns. In the natural motion case (Figure 8a), the torso trajectory exhibits a regular and repeatable oscillatory pattern with consistent range extrema and smooth transitions between seated and standing positions. This reflects a stable and rhythmic STS execution with minimal inter-cycle variability.

The pushing-off-knees condition (Figure 8b) was deliberately simulated by performing STS transitions with hands placed on the knees during the ascent phase, while keeping all other movement parameters identical to the natural condition (approximately 2 s per phase, yielding a full-cycle duration of approximately 4 s). This controlled design was intentional: by isolating the effect of upper-limb placement on the radar signature while keeping gross torso kinematics unchanged, the simulation allows direct attribution of any RTI signature differences to the additional hand reflector rather than to changes in movement speed or amplitude. As expected from this design, temporal and kinematic summary features including cycle duration, ascent and descent durations, and peak velocity, remained comparable to the natural condition. However, examination of the RTI map reveals a broader and more diffuse signal distribution at the seated position in the knee push-off condition compared to natural STS, consistent with the presence of an additional reflector contributed by the hands placed on the knees. This broader signature suggests that radar is sensitive to upper-limb involvement even when gross torso kinematics are preserved, providing a qualitative indicator of compensatory hand use. For a more ecologically valid simulation in which the participant genuinely relies on upper-limb assistance to rise, several candidate quantitative discriminators could be proposed. Trajectory asymmetry, captured by the ascent-to-descent duration ratio $T_{\mathrm{ascent}}/T_{\mathrm{descent}}$ and velocity ratio $\rho_{v}$, would be expected to deviate from unity when upper-limb assistance prolongs the rising phase relative to descent. Spectral entropy of the range trajectory would increase in the presence of two reflectors with partially independent motion, reflecting reduced signal periodicity compared to single-reflector natural STS.

For the motion speed variability condition (Figure 8c), the RTI map reveals irregular spacing between successive STS cycles and noticeable fluctuations in trajectory amplitude. The speed variability condition was simulated by performing repeated STS transitions in a fixed sequence of fast, normal, and slow executions. Sub-motion durations were selected based on the participant’s natural STS speed, measured at approximately 1.0 s per phase, with fast and slow variants set to 0.5 s and 2.0 s respectively, yielding full-cycle durations of approximately 1 s, 2 s, and 4 s for fast, normal, and slow executions. To quantify this variability, the spectral bandwidth $BW_{3\;\mathrm{dB}}$ was extracted from the dominant torso range trajectory and compared against the three constant-speed conditions. The speed variability trial yielded $BW_{3\;\mathrm{dB}}=0.0149$ Hz, which is approximately twice the bandwidth of the fastest constant-speed trial (0.0076 Hz, Table 5), reflecting the spread of spectral energy across the multiple cadences present in the mixed-speed execution pattern. The dominant spectral period of 7.05 s is consistent with the macro-periodicity of the repeating fast–normal–slow sequence, as the total duration of one complete pattern repetition is approximately 1+2+4=7 s. Per-cycle temporal feature extraction was not pursued for this condition, as the fast sub-motion durations (≈0.5 s per phase) approach the temporal resolution of frame-level radar processing at ∼13 Hz (one range profile per 77 ms frame interval), which provides approximately six samples per fast phase and is insufficient for reliable detection of seated and standing positions within each cycle. Radar systems with higher frame rates would provide sufficient temporal resolution for per-cycle feature extraction of fast compensatory movements and are identified as a direction for future work. Collectively, these results demonstrate that $BW_{3\;\mathrm{dB}}$ provides a compact and sensitive spectral signature for distinguishing mixed-speed from constant-speed STS execution, with wider bandwidth reflecting greater spectral energy spread across multiple execution cadences.

The rocking condition (Figure 8d) is characterized by pronounced forward–backward torso displacement prior to full standing. This behavior produces additional oscillatory components in the RTI map and a less distinct separation between seated and standing states. The resulting trajectory shows increased complexity and variability, consistent with preparatory rocking used to generate momentum before standing.

Overall, these RTI patterns demonstrate that abnormal STS behaviors introduce measurable distortions in both the temporal structure and spatial trajectory of the torso motion. Compared to natural transitions, abnormal motions exhibit increased variability, altered ascent dynamics, and reduced movement regularity.

**Figure 9:**
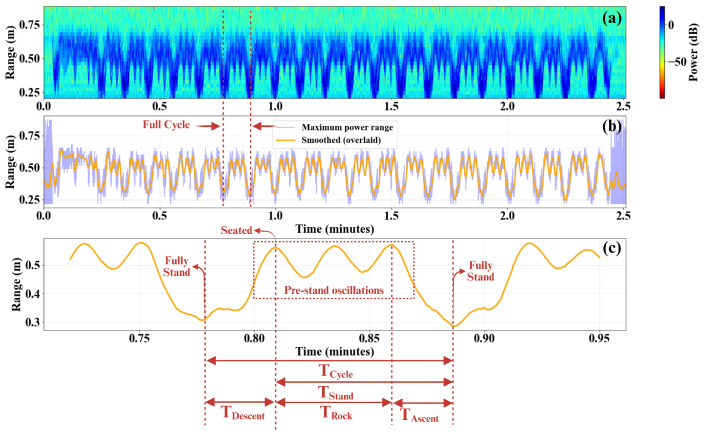
Detailed temporal characterization of a rocking STS transition. (**a**) RTI map illustrating repeated STS cycles with preparatory oscillations prior to standing. (**b**) Extracted maximum-power range trajectory with Savitzky–Golay smoothing, highlighting a representative full cycle. (**c**) Zoomed view of a single rocking cycle showing the definitions of $T_{\mathrm{cycle}}$, $T_{\mathrm{stand}}$, $T_{\mathrm{descent}}$, $T_{\mathrm{rock}}$, and $T_{\mathrm{ascent}}$. The rocking phase is characterized by multiple seated peaks between two consecutive fully standing positions.

To further quantify the preparatory rocking behavior, Figure 9 presents a detailed temporal decomposition of a representative rocking STS cycle. The RTI map in Figure 9a shows repeated oscillatory activity preceding the final standing transition. The extracted and smoothed range trajectory in Figure 9b highlights the full-cycle structure, while Figure 9c illustrates the subdivision of the cycle into $T_{\mathrm{descent}}$, $T_{\mathrm{rock}}$, $T_{\mathrm{ascent}}$, and $T_{\mathrm{stand}}$ components.

Unlike natural STS transitions, which exhibit a single seated peak between two standing events, rocking trials contain multiple intermediate seated peaks prior to the final rise. As a result, the standing transition duration $T_{\mathrm{stand}}$ is decomposed into a preparatory rocking interval $T_{\mathrm{rock}}$ and a final ascent interval $T_{\mathrm{ascent}}$. This decomposition enables isolation of the momentum-generating oscillatory phase from the actual rise-to-stand movement.

The quantitative statistics of these temporal parameters are summarized in Table 6, demonstrating that rocking behavior substantially increases total cycle duration and introduces measurable variability within the standing phase.

## 5. Conclusions

This study presented a feasibility demonstration of a non-contact, privacy-preserving radar framework for quantitative sit-to-stand (STS) transition analysis using a 60 GHz FMCW sensor in a residential environment. A signal-processing pipeline was developed to convert raw intermediate-frequency radar data into range–time intensity maps and extract temporal, kinematic, and spectral features characterizing STS motion without wearables or vision-based systems.

Three main findings emerged from this work. First, evidence-based design guidance was established for radar deployment: subject-facing, mid-torso mounting produced the most continuous and separable STS signatures, while side-facing placement and extreme mounting heights reduced effective radial motion or introduced clutter and shadowing artifacts. Second, radar-derived temporal and kinematic features captured systematic biomechanical scaling across execution speeds with high repeatability, with mean full-cycle duration ranging from 7.98 s (fast) to 23.90 s (slow), peak ascent velocity scaling from 0.311 m/s to 0.358 m/s, and standard deviations remaining low across 30 repeated cycles, confirming the consistency of both movement execution and the radar-based feature extraction pipeline. Third, simulated compensatory behaviors produced distinct and quantifiable trajectory deviations: preparatory rocking introduced a measurable oscillatory phase of mean duration 2.36 s, prolonging the total standing transition to 4.80 s, while knee push-off and speed variability produced characteristic RTI map distortions differentiable from natural motion. These findings were obtained with a single healthy adult participant under controlled conditions, establishing proof-of-concept for the proposed framework. Future work should extend validation to larger and clinically diverse cohorts, including older adults with mobility impairments, chronic pain, or neurodegenerative conditions, where radar-derived STS metrics hold particular promise as unobtrusive digital biomarkers of functional decline and pain-related compensatory behavior.

## Figures and Tables

**Table 1:** Summary of radar-derived STS features and their clinical relevance.

Feature Category	Specific Metric	Clinical Relevance
Temporal	Phase duration (s)	Overall mobility, endurance, and functional timing
Kinematic	Peak and mean radial velocity (m/s)	Lower-limb power and movement effort
Spectral	Dominant STS cycle frequency (Hz)	Movement rhythmicity and steadiness
Behavioral	Trajectory variability and rocking patterns	Identification of compensatory strategies and instability

**Table 2:** Radar configuration parameters used for STS data acquisition.

Parameter	Description	Value
Start Frequency (GHz)	FMCW chirp start frequency	58.0
End Frequency (GHz)	FMCW chirp end frequency	63.5
Bandwidth (GHz)	Frequency sweep bandwidth	5.5
Chirp Repetition Time (ms)	Time interval between consecutive chirps	0.591
Frame Repetition Time (ms)	Time interval between consecutive frames	77.27
ADC Sample Rate (MHz)	Analog-to-digital converter sampling rate	2.0
ADC Samples per Chirp	Number of ADC samples per chirp	64
Chirps per Frame	Number of chirps collected in one frame	64
Transmit Antennas	Number of active TX antennas	1
Receive Antennas	Number of active RX antennas	3
IF Gain (dB)	Intermediate frequency gain	23
Maximum Range (m)	Maximum unambiguous detectable range	1.75
Range Resolution (m)	Minimum resolvable distance	0.027
Maximum Velocity (m/s)	Maximum unambiguous radial velocity	2.09
Velocity Range (m/s)	Detectable radial velocity interval	[−2.09, 2.09]

**Table 3:** Temporal features extracted from 30 repeated sit-to-stand (STS) trials at different execution speeds (mean ± standard deviation).

Feature	Slow	Medium	Fast
$T_{\mathrm{cycle}}$ (s)	23.895±1.308	13.950±0.623	7.983±0.296
$T_{\mathrm{ascent}}$ (s)	3.113±1.490	2.226±0.728	1.649±0.895
$T_{\mathrm{descent}}$ (s)	3.618±1.074	2.539±0.481	1.512±0.978

**Table 4:** Kinematic features extracted from STS cycles at different execution speeds (mean ± standard deviation).

Feature	Slow	Medium	Fast
$D_{\mathrm{ascent}}$ (m)	0.3059±0.0271	0.2894±0.0279	0.2682±0.0272
$D_{\mathrm{descent}}$ (m)	−0.3364±0.0295	−0.3136±0.0374	−0.2812±0.0280
$V_{\mathrm{ascent},\mathrm{avg}}$ (m/s)	0.1291±0.0283	0.1481±0.0279	0.2115±0.0724
$V_{\mathrm{descent},\mathrm{avg}}$ (m/s)	−0.0943±0.0289	−0.1507±0.0293	−0.2372±0.0827
$V_{\mathrm{ascent},\mathrm{peak}}$ (m/s)	0.3108±0.0697	0.3239±0.0426	0.3582±0.0299
$V_{\mathrm{descent},\mathrm{peak}}$ (m/s)	−0.3047±0.0978	−0.3164±0.059	−0.3840±0.0499
Velocity ratio	0.9917	0.9830	1.046

**Table 5:** Spectral features extracted from repeated STS trials at different execution speeds.

Feature	Slow	Medium	Fast
$f_{\mathrm{peak}}$ (Hz)	0.0416	0.0712	0.1250
$BW_{3\;\mathrm{dB}}$ (Hz)	0.0027	0.0046	0.0076

**Table 6:** Temporal parameters extracted for the simulated rocking STS transitions (mean ± standard deviation).

Parameters	$T_{\mathrm{cycle}}$	$T_{\mathrm{stand}}$	$T_{\mathrm{descent}}$	$T_{\mathrm{ascent}}$	$T_{\mathrm{rock}}$
**Value (s)**	6.712±0.364	4.799±0.274	1.658±0.128	1.889±0.142	2.363±0.121

## Data Availability

The original contributions presented in this study are included in the article. Further inquiries can be directed to the corresponding authors.

## Abbreviations

The following abbreviations are used in this manuscript:

AD	Alzheimer’s disease
ADC	Analog-to-digital converter
FMCW	Frequency-modulated continuous-wave
FFT	Fast Fourier transform
ICC	Intraclass correlation coefficient
IF	Intermediate frequency
ML	Machine learning
MOBID-2	Mobilization-Observation-Behaviour-Intensity-Dementia-2
PAINAD	Pain Assessment in Advanced Dementia
RTI	Range–time intensity
SFR	Subject-facing radar
SNR	Signal-to-noise ratio
SSFR	Side-facing radar
STS	Sit-to-stand
